# Mandible osteoradionecrosis after high-dose radiation therapy for head and neck cancers: risk factors and dosimetric analysis

**DOI:** 10.2340/1651-226X.2024.35222

**Published:** 2024-05-06

**Authors:** Lars Merring-Mikkelsen, Mads Høyrup Brincker, Maria Andersen, Özlem Kesmez Yildirim, Martin Skovmos Nielsen

**Affiliations:** a Department of Medical Physics, Oncology, Aalborg University Hospital, Aalborg, Denmark; b Department of Oncology, Aalborg University Hospital, Aalborg, Denmark; c Department of Oral Surgery, Aalborg University Hospital, Aalborg, Denmark; d Department of Clinical Medicine, Aalborg University, Aalborg, Denmark

**Keywords:** Osteoradionecrosis, radiotherapy, head and neck, risk factors

## Background

High dose radiotherapy (RT) is a curative treatment for head and neck (HN) cancers. A possible late complication of this type of RT is osteoradionecrosis (ORN) in the mandible: a condition defined by exposed irradiated bone that fails to heal over a period of 3 months without any evidence of a persistent or recurrent tumour [1]. Onset of ORN may lead to a severe reduction in quality of life, and therefore all possible prophylactic and therapeutic measures of prevention should be made [2].

The existing literature presents inconsistent findings on the significance of patient-related factors like smoking and comorbidities such as osteoporosis and diabetes in the development of ORN, indicating a need for additional studies to draw conclusive results [3–8]. However, a consistently recognized determinant across studies is the dose to the mandible. While most clinical guidelines and protocols have generally dismissed doses under 50 Gy as contributing to ORN [9], more recent findings suggest that intermediate dose levels ranging from 30 to 50 Gy may indeed influence the risk of developing this condition [5, 6].

Looking back through the years, the incidence of ORN has decreased significantly, where older RT techniques such as 3-dimensional (3D) conformal fields have reported an incidence up to 20% [10]. Newer treatment techniques such as intensity modulated radiation therapy (IMRT) or volumetric modulated arc therapy (VMAT), make it possible to limit the dose to all organs at risk (OAR), such as the mandible. The use of IMRT has demonstrated a reduction in ORN incidence, although the literature reports varying occurrences ranging from 1 to 11% [3–6, 11–13].

A contributing factor to these varying results might be due to ORN being a late-onset complication, where the time between the last RT treatment and onset can differ significantly. However, the onset of ORN most often occurs between 4 months and 2 years but risk remains for life, although to a lesser degree [10]. Consequently, patients may have passed away before the onset of ORN, potentially leading to data misrepresentation.

This study aims to explore the correlation between the incidence of ORN and potential risk factors.

## Materials and methods

The inclusion criteria for patients were a histopathological confirmation of any type of HN cancer and a prescribed RT dose of 66 or 68 Gy in 2 Gy fractions. Patients receiving unilateral or bilateral treatment were both included in this study. All patients had a planning target volume (PTV) overlap with the mandible volume. Patients deceased within 1 year after their last RT treatment were excluded, because of the potential misrepresentation of data, since ORN is a late-onset complication. A total of 26 patients were excluded because of this criterion.

A cohort of 250 patients diagnosed with HN cancer and treated with high-dose RT from January 2018 to December 2021 were found and 10 patients of the 250 patients were identified with ORN.

After RT ended, patients had a follow-up procedure in accordance with The Danish Head and Neck cancer Study Group’s (DAHANCA) guidelines [14]. All patients were told to contact the clinic, if any new discomforts occur.

The patients’ medical data were extracted from their individual clinical journals. The data extracted were gender, age at treatment, potential onset date of ORN, diabetes, osteoporosis, and smoking history. Smoking history was categorized into two groups: those who have smoked or are currently smoking and those who have never smoked.

Patients with a record of receiving any form of bisphosphonates were categorized as having osteoporosis. To evaluate the dose to the mandible, the patients had their corresponding treatment plans manually extracted from the treatment planning system Aria. Treatment plans were made based on a planning CT scan of the patients prior to their treatment, utilising a Siemens SOMATOM go.Open Pro CT scanner. Dose constraints and outlining of targets and OAR were in accordance with DAHANCA’s guidelines [14]. The treatment planning and delivery utilised 6 MV photons with either IMRT or VMAT. All patients were immobilised in a thermoplastic mask and treated with a Varian linear accelerator (LINAC). The main content extracted from the treatment plans was the Dose-Volume Histogram (DVH) of the mandible. Each DVH had a step size of 0.1 Gy and was analysed using MATLAB^®^.

Patients with or without ORN were separated into two groups and an average DVH in relative and absolute volume was found for each group. A paired *t*-test was used to analyse the average DVHs of each group. The chi-squared test was used to identify the potential risk factors for each group. Furthermore, univariable Cox regression was performed to calculate the hazard ratio (HR) for each potential risk factor.

Permission to record and handle data was granted by the North Denmark Region (eDoc: 2021–000452-255).

## Results

The characteristics of the patients included in this study are shown in Table 1. There was a significant difference in smoking history between the two groups (*p* < 0.05), where patients with ORN were more often smokers.

**Table 1:** Characteristics of the 10 patients with ORN and the 240 control patients.

Characteristic	ORN (%)	Non-ORN	*P*
**Gender**			0.83
** Male**	8 (80.0)	185 (77.1)	
** Female**	2 (20.0)	55 (22.9)	
**Median age at RT (years, range)**	59.7 (46–67)	63.4 (40–91)	0.11
**Smoking history**			0.036
** Yes**	10 (100)	166 (69.2)	
** No**	0 (0)	74 (30.8)	
**Diabetes mellitus**			0.31
** Yes**	2 (20.0)	24 (10.0)	
** No**	8 (80.0)	216 (90.0)	
**Osteoporosis**			0.11
** Yes**	2 (20.0)	16 (6.7)	
** No**	8 (80.0)	224 (93.3)	
**D_mean_ of Mandible (Gy)**	40.7	32.3	0.003

ORN: osteoradionecrosis; RT: radiotherapy.

Of the 250 patients included 10 developed ORN, resulting in an incidence of 4%. The average time to event from the last treatment were 16.7 months (range 1–46) where the median was 6 months.

**Figure 1:**
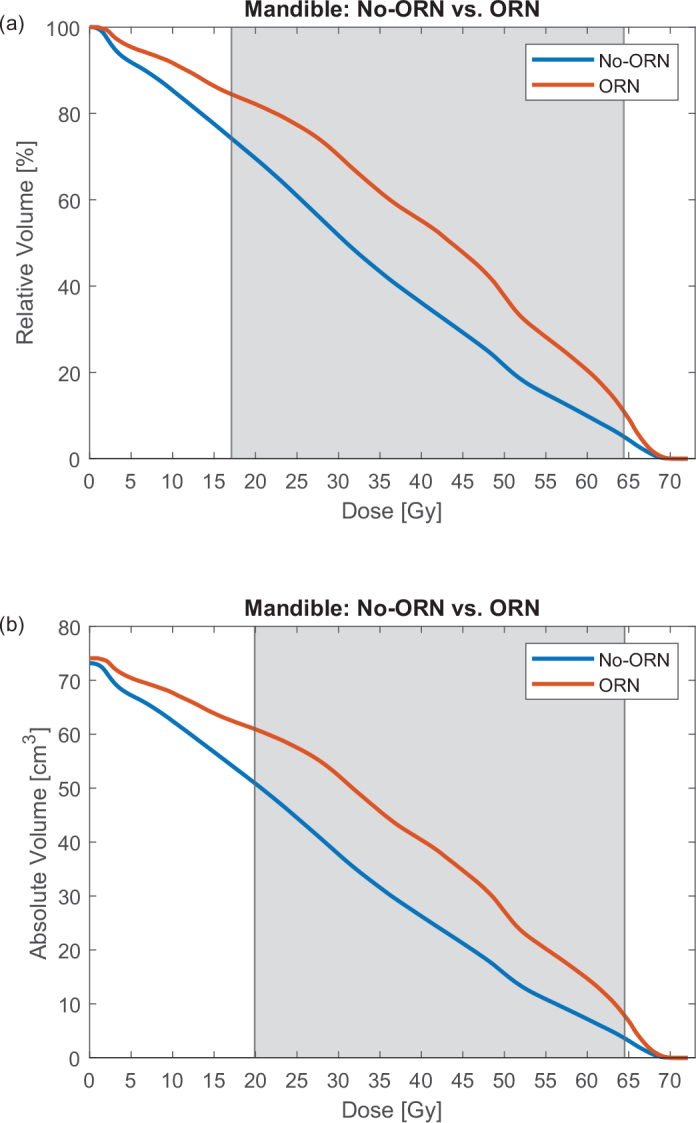
Two figures showing the mean DVH for the two groups, where the orange line represents patients with ORN and the blue line represents non-ORN patients. For (a) the y-axis is plotted in relative volume and for (b) the y-axis is plotted in absolute volume. The grey area indicates where the two curves are statistically different (p < 0.05). ORN: osteoradionecrosis.

A significant difference in the mean mandible dose for the two groups was found, namely 40.7 Gy (range 32.2–48.6) for patients with ORN and 32.3 Gy (range 14.2–56.2) for patients without ORN (*p* = 0.003). Figure 1 shows the average DVH for the two groups, plotted as absolute and relative volumes. The grey area in Figure 1 shows where the two curves are significantly different (*p* < 0.05), with 17.1–64.4 Gy in relative volume and 19.9–64.5 Gy in absolute volume.

The results of a univariable Cox regression for potential risk factors for developing ORN are presented in Table 2. Smoking is not included due to the lack of non-smokers developing ORN, making the Cox regression analysis unfeasible. The only significant risk factor for the patient cohort, was the mean dose to the mandible being over 40 Gy (HR 5.93; CI 1.27–27.8).

**Table 2:** Univariable analysis of potential HR for the development of ORN.

Univariable analysis	HR	95% CI	*P*
**Gender**	1.20	0.28–5.23	0.81
**Diabetes mellitus**	2.15	0.28–16.4	0.32
**Osteoporosis**	3.28	0.29–36.7	0.11
**D_mean_ > 40 Gy**	5.93	1.27–27.8	0.002

HR: hazard ratio; ORN: osteoradionecrosis.

## Discussion

The incidence of developing ORN was found to be 4%, which is in accordance with other studies finding an incidence of 3–6%, using modern RT treatment methods such as IMRT and having larger patient cohorts [12–15]. The same studies showed a large variation in the time to onset of ORN, ranging from 1 month to over a decade. This study found the average time to onset of ORN to be 16.7 months, which is in accordance with other publications that found an average time between 8 and 19 months [5, 6].

It should be noticed that because ORN is a late-onset complication from RT, where time to event has been observed occurring 17 years after the last treatment day, there could still be some patients in this study’s cohort who will develop ORN eventually or have passed away before a potential onset [10]. This might lead to a misrepresentation of the data for this study. However, it has been observed in other studies, that 74–90% of ORN cases develop within the first 3 years, meaning it is likely only a few patients who are misrepresented [5, 10].

The only statistically significant difference between patients with and without ORN was mean dose to the mandible and smoking, as shown in Table 1. The significant difference in mean dose to the mandible is reflected in the interval from 20 to 60 Gy, as can be seen in Figure 1. The impact of smoking on developing ORN has shown various results in the literature. For instance, Aarup-Kristensen et al., using a similar grading system of smoking as this study, but with a larger patient cohort, observed only a marginal tendency for smoking to significantly impact ORN development [6]. In contrast, Tsai et al.’s study, which utilized a more detailed grading system for smoking status (Never, Former, and Current), found a significant difference, particularly among patients who continued smoking after treatment [3]. These findings show the importance of a comprehensive smoking grading system in assessing its role for the development of ORN.

The literature for determining the impact of osteoporosis on the development of ORN is scarce. Table 2 shows a slight tendency (not significant) towards osteoporosis being a risk factor for developing ORN. A study carried out by Miniello et al. showed no clear tendency towards developing ORN earlier for ORN patients receiving bisphosphonates compared with ORN patients receiving no bisphosphonates [7]. More long-term prospective studies are needed to fully understand the impact of osteoporosis on the development of ORN.

Diabetes mellitus shows no statistical correlation or hazard towards the development of ORN, as shown in Tables 1 and 2, which has also been seen in other studies [4, 8].

Comparing all potential risk factors for ORN in this study, dose seems to be the most determining factor. There is a clear distinction in the mean dose to the mandible between the groups and a HR of 5.93 for patients with a mean dose above 40 Gy. The impact of dose on the development of ORN is well known in literature, however no single determining factor has yet been deducted [5, 6, 8]. Local dose in areas where ORN has developed could be interesting to investigate, however it is out of the scope of this study but should be considered in future studies.

To our knowledge, no other study has analysed the dosimetric characteristics of the mandible in terms of relative and absolute volumes. Only analysing the DVH in relative volume should not be a given, because the volume of the mandible varied from 32.2 to 112.8 cm^3^ for patients in this study. This can mask a potential volumetric effect if the DVH is only analysed in relative volume. The significant difference between the two curves in Figure 1 starts at 17.1 Gy for relative volume and 19.9 Gy for absolute volume, and both end roughly at the same dose. It would be more correct to always analyse the DVH in absolute volume but due to the small difference between the areas of statistical difference, most studies would find it sufficient to analyse the DVH only in relative volume. The average mandibular volume of patients with ORN is comparable to that of patients without ORN (see Figure 1b), both closely aligning with the mean mandibular volume of the entire cohort, which is 73.4 cm³ (SD 14).

For doses above 65 Gy (see Figure 1), no significant impact on the development of ORN was observed, which is in accordance with other studies only seeing a significant difference from 30 to 60 Gy [5, 6]. Therefore, a larger focus should be put on reducing the dose between 20 and 60 Gy and possibly also implement new dose restriction criteria into treatment guidelines. This is also suggested by the MD Anderson Head and Neck Cancer Symptom Working Group and others [5, 9]. In turn, this will also reduce the mean dose to the mandible and therefore reduce the potential HR for ORN.

One could fear that reducing the dose to the mandible would increase the dose to the surrounding OARs. A study conducted by Rulach et al. [16] implemented multicriteria optimisation for lung cancer treatment and saw a total improvement in dose to all OARs except for one dosimetric parameter of the heart. Looking at Figure S1 and Table S1, the dose to the mandible and surrounding OAR was significantly reduced from 2018 to 2021 for the patients in this study, demonstrating the possibility of reducing the dose to all OARs simultaneously. This reduction in dose to the mandible will likely result in a smaller incidence of ORN in the coming years, because dose is prominent risk factor for developing ORN. Other studies have observed a drop in the incidence of ORN from 21 to 5% after using IMRT instead of 3D-conformal RT [10, 14].

In conclusion, this study investigated potential risk factors associated with the development of ORN in patients treated for HN cancers with high-dose RT. Smoking and mandible mean dose were found to be significant risk factors for the development of ORN.

## Supplementary Material

Mandible osteoradionecrosis after high-dose radiation therapy for head and neck cancers: risk factors and dosimetric analysis
