# Supplementary material for: Mandible osteoradionecrosis after high-dose radiation therapy for head and neck cancers: risk factors and dosimetric analysis
Source: Acta Oncol. 2024 May 6;63:35222. doi: 10.2340/1651-226X.2024.35222 (PMC11332527; doi:10.2340/1651-226X.2024.35222)
Supplement: Mandible osteoradionecrosis after high-dose radiation therapy for head and neck cancers: risk factors and dosimetric analysis [file AO-63-35222-s1.pdf]

Supplementary material has been published as submitted. It has not been copyedited or typeset by Acta Oncologica.

Table S1: Average mean dose and confidence interval for each OAR per year.

Average Mean Dose (Gy)	2018	2019	2020	2021
**Mandible**	36.4 ±2.1	33.5 ±1.6	32.9 ±2.2	27.9 ±2.1
**Oral Cavity**	41.2 ±2.6	37.3 ±2.4	35.4 ±3.2	30.9 ±2.0
**Parotids**	32.8 ±1.9	30.7 ±2.2	28.2 ±2.3	24.7 ±2.2
**Submandibularis**	55.3 ±2.6	54.3 ±2.5	50.9 ±2.8	47.4 ±3.1
